# Clinical Experience of Sirolimus Regarding Efficacy and Safety in Systemic Lupus Erythematosus

**DOI:** 10.3389/fphar.2019.00082

**Published:** 2019-02-06

**Authors:** Per Eriksson, Philip Wallin, Christopher Sjöwall

**Affiliations:** Rheumatology/Division of Neuro and Inflammation Sciences, Department of Clinical and Experimental Medicine, Linköping University, Linköping, Sweden

**Keywords:** sirolimus, arthritis, tendinitis, musculoskeletal pain, quality-of-life, systemic lupus erythematosus

## Abstract

New treatment options constitute unmet needs for patients diagnosed with systemic lupus erythematosus (SLE). Inhibition of the mammalian target of rapamycin (mTOR) pathway by sirolimus, a drug approved and in clinical use to prevent transplant rejection, has shown promising effects in lupus animal models as well as in patients with both antiphospholipid syndrome and SLE. Sirolimus inhibits antigen-induced T cell proliferation and increases the number of circulating regulatory T cells. Recently, sirolimus was tested in an open label phase 1/2 trial, including 43 patients with active SLE, resistant or intolerant to conventional medications. The results were encouraging showing a progressive improvement, including mucocutaneous and musculoskeletal manifestations. At our university unit, we have more than 16 years’ experience of sirolimus as treatment for non-renal manifestations of SLE. Herein, we retrospectively evaluated data on tolerance, dosage, affected organ systems, disease activity measures, corticosteroid reduction, concomitant immunosuppressive therapies, and patient-reported outcome measures (PROMs) such as pain intensity, fatigue, well-being and quality-of-life (QoL) in 27 Caucasian patients with mildly active SLE. Musculoskeletal manifestation was the main reason for sirolimus treatment followed by skin involvement and leukocytopenia. Mean time on sirolimus was 47.1 (range 2–140) months. Decreasing global disease activity was observed, as measured by the clinical SLE disease activity index-2000, with a mean reduction of 2.5 points (range -10 to 0) and a corresponding mean reduction of the physician’s global assessment (0–4) of 0.64 (range -2 to 0). The mean daily dose of corticosteroids (prednisolone) was reduced by 3.3 mg (-12.5 to 0). Non-significant trends toward improvements of QoL and pain intensity were found. Serious side-effects were not seen during sirolimus treatment, but early withdrawal due to nausea (*n* = 4) and non-serious infections (*n* = 2) appeared. This observational study, including longtime real-life use of sirolimus in SLE, is the largest to date and it essentially confirms the results of the recent phase 1/2 trial. Our data indicate that sirolimus is efficient in patients with musculoskeletal SLE manifestations, particularly arthritis and tendinitis. Further randomized controlled trials evaluating the potential benefits of sirolimus in SLE are warranted, but should aim to enroll patients with shorter disease duration, less accrued damage, and more diverse ethnicities.

## Introduction

Novel therapies aiding patients with systemic lupus erythematosus (SLE) constitute an unmet need since the available drugs often are limited to efficacy in certain disease phenotypes, and may have significant side-effects (Lateef and Petri, 2012). In fact, only few of the medications used in clinical practice today are approved for SLE, and several new candidate drugs have recently failed to meet their primary end-points in randomized controlled trials (Doria et al., 2017; Geh and Gordon, 2018). Instead, current therapeutic strategies for SLE mainly rely on clinical experience of older therapies used in other rheumatic conditions, or originate from the area of transplantation.

The pathogenesis of SLE is multifactorial. Genetic susceptibility and environmental factors play important roles and are accompanied by the involvement of T and B cells, dendritic cells, macrophages and neutrophils (Bengtsson and Rönnblom, 2017). The profound T cell dysfunction found in SLE has partly been attributed to activation of the mammalian target of rapamycin (mTOR), representing an intracellular serine/threonine receptor which regulates cell growth, proliferation and survival. mTOR is formed by a protein complex which includes mTORC1 and mTORC2 (Perl, 2016). mTORC1 drives the expansion of T helper (Th) type 1 cells, Th17 T cells, and CD4-CD8- (double-negative) T cells. mTORC2, as well as mTORC1, inhibit the development of CD4+CD25+FoxP3+ T regulatory cells. In addition, the differentiation of macrophages and dendritic cells is influenced by mTOR (Thomson et al., 2009; Perl, 2016).

Mammalian target of rapamycin is thus implicated in the pathogenesis of SLE in several ways. Patients with SLE have a reduced number of regulatory T cells (T_regs_) with impaired suppressive activity (Banica et al., 2017; Kato and Perl, 2018). Follicular helper T (Tfh) cells are critical for germinal center formation and B cell activation. Tfh cells are expanded in SLE, and mTOR1 may be of importance for Tfh differentiation, although results are conflicting (Oaks et al., 2016). Furthermore, the B cell stimulating factor BAFF promotes B cell activation via mTOR activation (Ke et al., 2014), and inhibition of mTOR in plasmacytoid dendritic cells limits production of type I interferons, which has obvious implications for SLE (Cao et al., 2008; Bengtsson and Rönnblom, 2017).

Rapamycin, under the generic designation sirolimus, is a drug approved and in clinical use to prevent transplant rejection. Rapamycin has been shown to prevent the development of nephritis in lupus-prone mice (Warner et al., 1994; Lui et al., 2008). Just recently, sirolimus was tested in an open label phase 1/2 trial, including 43 patients with active SLE, resistant or intolerant to conventional medications. The results were encouraging showing a progressive improvement in several disease phenotypes, including mucocutaneous and musculoskeletal manifestations (Lai et al., 2018). In addition, involvement of the mTOR pathway in vascular lesions associated with the antiphospholipid syndrome (APS) has also been suggested and may be of high relevance also in SLE (Canaud et al., 2014). In a recently published case series of 16 patients with active or quiescent lupus nephritis where 7 had a previous history of malignancy, Yap and co-authors described an auspicious response to sirolimus treatment (Yap et al., 2018). However, to our knowledge, larger compilations on longterm real-life experience of sirolimus in SLE have so far not been reported.

At our university unit, we have more than 16 years’ experience of sirolimus as treatment for non-renal manifestations of SLE. Herein, we systematically evaluated our retrospective sirolimus data in relation to tolerance, dosage, affected organ systems, disease activity measures, corticosteroid reduction, concomitant immunosuppressive therapies, and patient-reported outcome measures (PROMs). Reduction of global SLE disease activity as defined by the physician’s global assessment (PGA) and the clinical SLE Disease Activity Index 2000 (cSLEDAI-2K) score (Scott, 1993; Uribe et al., 2004) constituted the primary outcome of the present study.

## Materials and Methods

### Patients

The University Hospital in Linköping constitutes a tertiary referral center serving two other regional public hospitals in the county council of Östergötland, Sweden. At the Rheumatology outpatient clinic, we have long experience of monitoring patients with SLE by a prospective, structured follow-up program “KLURING” (Swedish acronym for *Clinical LUpus Register In Northeastern Gothia*), including registration of disease phenotypes, ongoing medication, and comorbidities (Frodlund et al., 2013).

As part of the KLURING cohort, a total of 27 patients with SLE, classified according to the 1982 American College of Rheumatology (ACR) criteria (Tan et al., 1982; Ighe et al., 2015), received sirolimus in daily doses of 1–3 mg between June 2002 and August 2018, and were followed from initiation of treatment until withdrawal, death or end of study period. All patients had previously been intolerant, or were judged as inadequate responders, to at least two disease-modifying anti-rheumatic drugs (DMARDs). Patient characteristics at the initiation of sirolimus treatment are further detailed in Table 1.

**Table 1:** Characteristics of the included patients at the start of sirolimus treatment.

Patient characteristics	Mean (range) or %
	All (*n* = 27)
**Background variables**	
Females	100
Age (years)	44.3 (20–65)
Duration of SLE (years)	9.8 (2–34)
Weight (kg)	65.6 (47–93)
Length (cm)	165.7 (147–176)
Caucasian ethnicity	100
cSLEDAI (score)	4.5 (1–12)
PGA (score)	1.3 (0–2)
SDI (score)	1.0 (0–6)
Number of fulfilled ACR criteria	5.5 (4–8)
**Concomitant medication**	
Prednisolone, daily dose (mg)	7.5 (0–20)
Hydroxychloroquine	59.2
Methotrexate	7.4
Mycophenolate mofetil	11.1
Warfarin	14.8
Acetylsalicylic acid	29.6
Statins	0
**Clinical phenotypes (ACR-82 definitions)**	
(1) Malar rash	48.1
(2) Discoid rash	18.5
(3) Photosensitivity	63.0
(4) Oral ulcers	22.2
(5) Arthritis	100
(6) Serositis	48.1
(a) Pleuritis	48.1
(b) Pericarditis	3.7
(7) Renal disorder	25.9
(8) Neurologic disorder	3.7
(a) Seizures	3.7
(b) Psychosis	0
(9) Hematologic disorder	66.7
(a) Hemolytic anemia	3.7
(b) Leukocytopenia	37.0
(c) Lymphocytopenia	44.4
(d) Thrombocytopenia	14.8
(10) Immunologic disorder	51.9
(a) Anti-dsDNA antibody	44.4
(b) Anti-Smith antibody	7.4
(11) IF-ANA	100


*ACR, American College of Rheumatology; IF-ANA, immunofluorescence microscopy antinuclear antibodies; cSLEDAI-2K, clinical Systemic lupus erythematosus disease activity index 2000; SDI, SLICC/ACR damage index*.

### Assessments

Systemic lupus erythematosus disease activity was assessed by the use of PGA (graded 0–4) (Scott, 1993) and the cSLEDAI-2K score (which excludes items for low complement and positive anti-dsDNA antibodies) (Uribe et al., 2004). Acquired organ damage, required to have been persistent for at least 6 months, was recorded by the Systemic Lupus International Collaborating Clinics (SLICC)/ACR damage index (SDI) encompassing damage in 12 defined organ systems (Gladman et al., 1996). Continuous data on PROMs were collected. The PROMs included data on quality-of-life (QoL) captured by the EQ-5D score (Leidl, 2009), functional ability estimated by the health assessment questionnaire (HAQ) (Lomi et al., 1995), as well as pain intensity, fatigue and well-being, all measured using the visual analog scale (VAS; graded 0–100 mm) (Hallert et al., 2003).

### Laboratory Measurements

Safety was continuously monitored by blood cell counts, liver enzymes (including alanine aminotransferase and aspartate aminotransferase), plasma creatinine, and blood lipids (including total cholesterol and triglycerides). Inflammatory and serological disease activity were followed by the erythrocyte sedimentation rate (ESR), and plasma analyses of C-reactive protein (CRP), creatine phosphokinase (CK), complement protein 3 (C3), and 4 (C4).

### Statistics

The GraphPad software (version 4.0; GraphPad Software Inc., San Diego, CA, United States) and the Python Language Reference (version 3.7, available at http://www.python.org, Python Software Foundation, Wilmington, DE, United States) were used for preparing figures and for statistical evaluation. Since the number of observations was different between many visits, repeated paired *t*-tests were used to examine differences in laboratory variables overtime and Wilcoxon’s test for paired samples was used to evaluate disease activity scores. Correlation analysis was performed using Pearson’s correlation coefficient. Two-tailed *p* < 0.05 was considered significant.

## Results

### Patients Treated With Sirolimus

As demonstrated in Table 2, 27 unique female SLE patients at our unit were prescribed sirolimus between June 2002 and August 2018 (study period). The mean daily dose was 1.5 mg (range 1–3). Before start of sirolimus, the mean number of failed DMARDs was 3.6 (range 2–6). The mean time on sirolimus was 47.1 (range 2–140) months. Six of 27 (22%) withdraw the drug due to nausea (*n* = 4) and non-serious infections (*n* = 2) before the 3-month evaluation visit (early cessation indicated by asterisks in Table 2), which was the reason why these six cases were excluded from efficacy analyses. At the last follow-up in August 2018, seven patients were still on treatment with sirolimus, and one individual (who had reached remission after 70 months on sirolimus) was not considered in need of the drug anymore; this corresponds to a drug survival of 38% regarding cases that passed the 3-month evaluation visit.

**Table 2:** Individual descriptions of the 27 female pations.

Patient number	Age at start (years)	Target organs	Sirolimus exposure (months)	Daily dose (mg) of sirolimus	Combining DMARDs	Cause of cessation	Number of DMARDs ahead of sirolimus	SDI at start	SDI at last follow-up on sirolimus
1	58	Musculoskeletal, leukocytopenia	117	2	HQ	Treatment ongoing	2	1	1
2	65	Musculoskeletal, discoid lupus	10	3	None	Rash, swelling of legs	3	6	6
3	43	Musculoskeletal	18	1	HQ, MMF	Decreasing effect	6	3	3
4	53	Musculoskeletal	96	1	None	Treatment ongoing	4	0	1
5	62	Musculoskeletal	35	2	HQ, MTX	Itching, headache	5	1	3
6^∗†^	51	Musculoskeletal, leukocytopenia	3	1	None	Infection, lack of efficacy	6	2	2
7	59	Musculoskeletal, malar rash	13	1	HQ	Increased liver enzymes	3	0	1
8	37	Musculoskeletal, alopecia, pleuritis	4	2	None	Nausea	5	1	1
9	27	Musculoskeletal	129	1	HQ	Treatment ongoing	4	0	2
10^†^	56	Musculoskeletal	31	2	None	Malignancy	3	1	1
11	35	Musculoskeletal	7	1	None	Itching, fatigue	2	1	1
12	61	Musculoskeletal	63	2	HQ	Treatment ongoing	2	1	2
13	44	Musculoskeletal	140	3	HQ, MMF	Treatment ongoing	4	1	1
14	52	Musculoskeletal	111	2	None	Treatment ongoing	3	1	2
15^†^	50	Musculoskeletal, malar rash	127	2	None	Infections	5	2	7
16^∗^	32	Musculoskeletal	2	1	HQ	Nausea	3	0	0
17	38	Musculoskeletal, photosensitivity	4	1	HQ	Lack of efficacy	4	0	0
18^∗^	48	Musculoskeletal	3	1	HQ	Nausea	2	0	0
19	20	Musculoskeletal	70	1	HQ	Reached remission	4	0	0
20	50	Musculoskeletal	104	1	HQ	Angioedema	4	0	1
21	48	Musculoskeletal	7	1	HQ, MMF	Nausea	3	1	1
22	21	Lupus profundus	27	1	HQ	Diarrhea	6	1	1
23^∗^	38	Musculoskeletal, malar rash	3	1	None	Nausea	2	0	0
24	34	Musculoskeletal, photosensitivity	135	1	HQ	Treatment ongoing	2	0	0
25^∗†^	44	Musculoskeletal	3	1	None	Infections	2	0	0
26^∗†^	29	Musculoskeletal, alopecia	2	2	MTX	Nausea	5	3	3
27	40	Musculoskeletal, oral/genital ulcers, acute cutaneous lupus	10	2	HQ	Lack of efficacy	5	0	0


*^∗^Early cessation (≤3 months). ^†^Deceased at the end of study period. HQ, hydroxychloroquine; MMF, mycophenolate mofetil; MTX, methotrexate*.

### Organ Manifestations

As shown in Table 2, musculoskeletal involvement was the target for sirolimus treatment (96%), followed by cutaneous lupus (37%), and leukocytopenia (7%). Regarding specific musculoskeletal manifestations, arthritis (54%) was the dominating reason for sirolimus, but tendinitis (15%) and arthralgia (31%) were also common. Seven of 27 (26%) patients had a history of renal involvement, but none had signs of active lupus nephritis at the initiation of sirolimus. 5 (19%) had concomitant APS. Sirolimus was frequently combined with corticosteroids, hydroxychloroquine (HQ) and/or other DMARDs as indicated in Table 2.

### Efficacy

**FIGURE 1:**
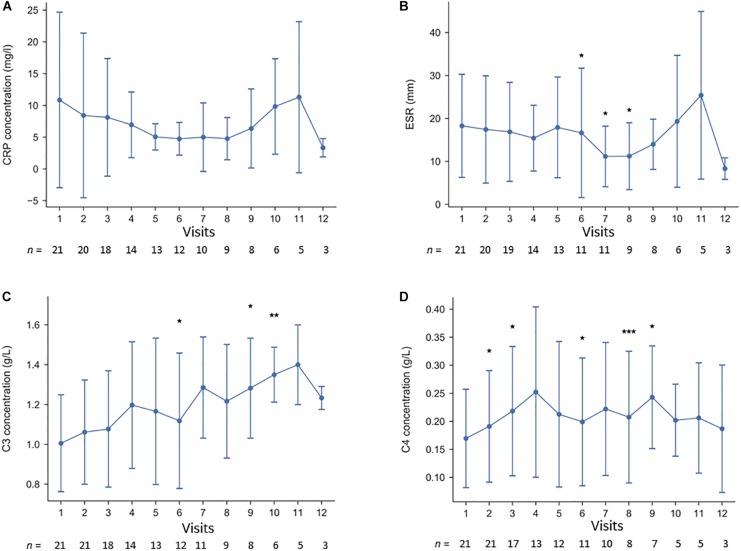
**(A–D)** Longitudinal laboratory efficacy data at the first 12 visits of the 21 cases that passed the 3-month evaluation visit; **(A)** C-reactive protein, **(B)** erythrocyte sedimentation rate, **(C)** complement protein 3, and **(D)** complement protein 4. ^∗^*p* < 0.05, ^∗∗^*p* < 0.01, and ^∗∗∗^*p* < 0.005.

**FIGURE 2:**
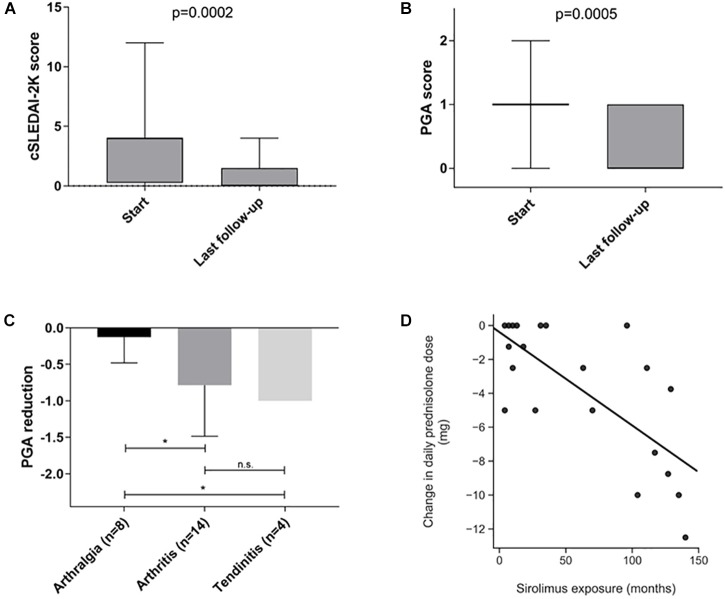
**(A–D)** Differences in global disease activity between start/initiation of sirolimus therapy with regard to **(A)** clinical SLEDAI-2K, and **(B)** physician’s global assessment (PGA). **(C)** Illustrates the reduction of PGA scores with regard to type of musculoskeletal manifestation. **(D)** Demonstrates the correlation between reduction of daily corticosteroid dose and the exposure of sirolimus in months. ^∗^*p* < 0.05.

Inflammatory and serological disease activity was followed over time by measurement of ESR, CRP, C3, C4, and CK. As illustrated in Figure 1, levels of C4 increased slightly over time, whereas CRP and ESR were rather stable. CK did not change significantly (not shown). As shown in Figure 2A, a decreased global disease activity was observed over time using cSLEDAI-2K (*p* = 0.0002) with a mean reduction of 2.5 (range -10 to 0) comparing the time-point of initiation with the last observation. A corresponding reduction of 0.64 (-2 to 0) regarding PGA (Figure 2B) was also found (*p* = 0.0005). Sirolimus appeared to be especially effective against arthritis and tendinitis, whereas patients with arthralgia did not respond (Figure 2C). The mean daily dose of corticosteroids (prednisolone) at start was 7.5 mg (Table 1), but it was reduced by 3.3 mg (range -12.5 to 0) comparing the time-point of sirolimus initiation with the last observation (*p* < 0.001). The correlation between exposure to sirolimus and reduction of prednisolone dose was highly significant (*r* = -0.7, *p* < 0.0004) (Figure 2D). No significant improvements of PROMs (EQ-5D, HAQ, VAS pain intensity/fatigue/well-being) were observed. SDI scores at the initiation, and at the time-point of last follow-up on sirolimus, are demonstrated in Table 2. As shown in Table 1, the mean SDI at initiation of sirolimus was 1.0 (0–6), and at last follow-up 1.5 (0–7). The mean annual accrual of SDI on sirolimus was 0.1 (range 0–0.9).

### Safety

At end of the study period, 22 of 27 cases were alive. The five deceased patients (mean age 53.4 years, range 33–63) had been on sirolimus for a mean time of 33.2 months (range 2–133). The cause of death was malignancy in three cases (adenocarcinoma of the lung, ovarian cancer, acute myeloid leukemia) of which two patients had early cessations of sirolimus (before the 3-month evaluation visit). Sepsis was the cause of death in the other two cases, whereof one patient had an early withdrawal. All five patients had discontinued sirolimus at the time-point of death, and none of the deaths were considered related to the drug. No renal flares, or onset of new lupus nephritis, were observed in any of the 27 patients.

No myocardial infarctions were registered, but two minor strokes were observed. Patient number 9, with SLE since 1983, was started on sirolimus because of arthritis in October 2007. Due to a new onset of seizure, a brain magnetic resonance imaging (MRI) was performed and showed new ischemic lesions. Antiphospholipid antibodies were detected and she was, in addition to SLE, diagnosed with APS which led to continuous treatment with warfarin. This event was indeed considered as an SLE exacerbation. However, the patient is still on sirolimus and has not had further strokes since then. Patient number 15, with multiple sclerosis since the 90s and SLE combined with APS since 2000, was started on sirolimus due to arthritis in June 2002. In 2009, she developed a minor warfarin-dependent cerebrovascular bleeding but the treatment with sirolimus was not discontinued until 2013.

**FIGURE 3:**
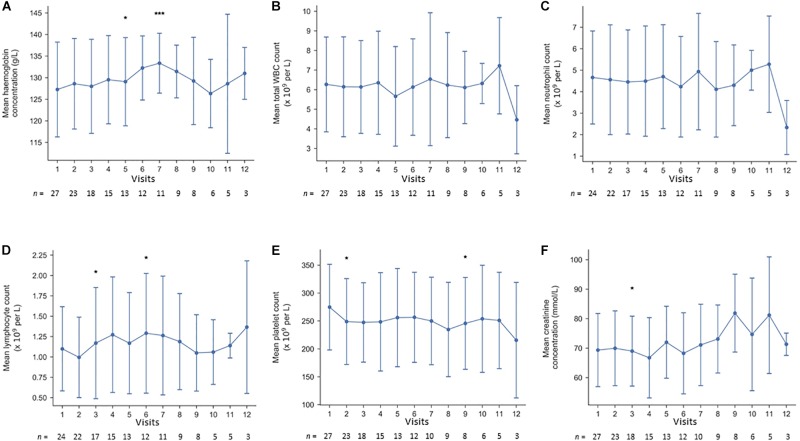
**(A–F)** Longitudinal laboratory safety data at the first 12 visits of the 27 cases; **(A)** hemoglobin, **(B)** white blood cell count, **(C)** neutrophil count, **(D)** lymphocyte count, **(E)** platelet count, and **(F)** plasma creatinine. ^∗^*p* < 0.05, ^∗∗^*p* < 0.01, ^∗∗∗^*p* < 0.005.

Drug safety was continuously monitored by blood tests. As demonstrated in Figure 3, no alarming signals regarding blood cell counts or renal function were noted. None of the patients developed hypercholesterolemia or triglyceridemia, leading to treatment with statins. After 13 months, patient number 7 ended treatment with sirolimus due to elevated liver enzymes. However, both alanine aminotransferase and aspartate aminotransferase normalized shortly after cessation.

## Discussion

Current knowledge on the performance of sirolimus in autoimmune diseases is increasing, but observational data are mainly missing in rheumatology. Thus, we aimed to retrospectively compile the 16-years of clinical experience we have at our university unit on sirolimus in SLE. As a drug with potency of blocking T cell activation, sirolimus has clear-cut implications for the SLE pathogenesis. In support of sirolimus as a suitable treatment option in SLE, blockade of the mTOR pathway has shown promising effects in lupus animal models (Warner et al., 1994; Bonegio et al., 2005; Lui et al., 2008) as well as in patients (Fernandez et al., 2006; Yap et al., 2012, 2018; Lai et al., 2013; Bride et al., 2016; Herold et al., 2018). The recent phase 1/2 trial showed effects primarily in the mucocutaneous and musculoskeletal organ systems (Lai et al., 2018). Reduced number of new episodes of rash was also reported but quite few patients developed cutaneous lupus over the study period why distinct conclusions were not possible. However, very interestingly, Lai et al. (2018) also observed that low levels of T_regs_ were reversed and high levels of interleukin (IL) 4 and IL-17 from other T cells than T_regs_ were reduced during the sirolimus treatment. Previously, Bride with coauthors reported beneficial effects of sirolimus on severe autoimmune cytopenias (Bride et al., 2016) and satisfactory response in individual patients with refractory discoid lupus erythematosus have been observed (Herold et al., 2018).

The Swedish healthcare system is tax funded and offers universal access, limiting the risks of patient selection bias, and drugs may be prescribed off label. As far as we know this observational study of sirolimus in SLE, including long time follow-up, is the largest to date. However, this is not a clinical trial and the included 27 patients with mildly active non-renal SLE were intolerant, or previously had an inadequate response, to at least two DMARDs. Some cases had tested multiple DMARDs without success and eventually failed on sirolimus as well. In addition, the study population was limited to cases without active renal or CNS involvement which is reflected by the rather low cSLEDAI-2K scores at start. Albeit, it is encouraging that none of our patients developed new (or incident) renal flares over the study period and the accrual of further damage was modest. Lack of longitudinal data on anti-dsDNA antibody levels and 28-joint disease activity scores constitute limitations of the study.

Comparing retrospective data with results from a clinical trial is challenging, but the outcome with reduced global disease activity (cSLEDAI-2K and PGA) essentially confirm the promising results of the phase 1/2 trial (Lai et al., 2018) and indicate that sirolimus is efficient in patients with musculoskeletal manifestations (i.e., arthritis and tendinitis). Whereas 2 mg sirolimus daily was used in the trial, we used slightly lower doses (mean 1.5 mg, with range 1–3 mg). Both dose regimens are lower than the doses usually prescribed in renal transplantation, a fact that has experimental support from lupus-prone mice (Warner et al., 1995).

The sirolimus trial did not include any PROMs (Lai et al., 2018). Although our patients with longtime follow-up showed trends toward improved QoL and less reported pain, the data were not statistically significant. The composition of the study population may be one of several reasons for this. Fifteen of 27 (56%) patients were already affected by irreversible organ damage (SDI>0), which has a proven impact on both QoL and activity limitations in SLE (Björk et al., 2015). Failure of up to 6 DMARDs before the initiation of sirolimus probably also led to a bias in term of selection of refractory cases. Thus, for future studies, there may be better options to record improvements on QoL and other PROMs if cases with more recent-onset SLE were eligible.

Premature atherosclerosis in SLE may be related to type I interferons, whereas traditional risk factors seem to be of less importance (Kahlenberg and Kaplan, 2013; Leonard et al., 2018). Thus, pharmacological intervention preventing vascular events in SLE would obviously be of interest. Sirolimus inhibits smooth muscle hypertrophy in vessel walls (Gallo et al., 1999; Elloso et al., 2003), which may outweigh the transient hyperlipidemia sometimes reported in transplanted patients treated with higher doses of sirolimus (Asleh et al., 2018). mTOR signaling is also the major pathway in inhibition of endothelial autophagy which is implicated in atherogenesis (Xiong et al., 2014). Furthermore, as concomitant APS occurs in approximately one third of SLE patients and the mTOR pathway is involved in the vascular lesions related to APS, sirolimus may be of high importance regarding future studies of vascular disease in SLE (Canaud et al., 2014). Our retrospective case series do not permit any conclusions concerning vascular disease, but on the other hand neither hypercholesterolemia nor triglyceridemia were observed among our patients taking low doses of sirolimus. Another action of sirolimus with important implications for lupus nephritis and its longtime prognosis is the anti-fibrotic effects, possibly mediated via E-cadherein in experimental renal fibrosis (Liu, 2006).

A non-negligible proportion of the patients (>20%) experienced non-serious side-effects or general discomfort and stopped sirolimus soon after its introduction. However, major side-effects were not seen and routine laboratory follow-up was normal in almost all cases. The rate of malignancies (11%) may appear high, but none of them occurred during sirolimus therapy and in two of the cases exposure to the drug was very short. A causative effect of sirolimus is unlikely but cannot be entirely excluded. The question is also hampered by the fact that the longterm risk of several types of cancers in SLE is increased (Bernatsky et al., 2013). Data from organ transplant recipients show that longterm immunosuppressive regimens which include mTOR inhibitors are associated with an overall reduced cancer risk when compared to patients not treated with mTOR inhibitors (Yanik et al., 2015). In transplantation, however, use of sirolimus has been associated with pneumonitis, microangiopathy, thrombocytopenia, hypercholesterolemia, liver toxicity, lymphangioleiomyomatosis, and increased proteinuria in nephrotic patients (Marti and Frey, 2005; Takada et al., 2016). None of the above appeared in our series.

## Conclusion

In summary, low doses of sirolimus were efficient in reducing global disease activity, especially regarding musculoskeletal manifestations, for patients with established mildly active SLE. Corticosteroids could be withdrawn or significantly reduced in many patients. Serious side-effects were not seen, although some patients stopped medication early due to non-serious discomfort. Only Caucasian patients were enrolled herein why it is warranted that further randomized controlled trials evaluating the potential benefits of sirolimus in SLE encompass larger and more mixed groups of cases.

## Ethics Statement

In Sweden, drugs are allowed to be used off label. Nevertheless, informed consent was obtained from all subjects. The research protocol was approved by the Regional Ethics Review Board in Linköping, Sweden (Decision No. M75-08/2008).

## Author Contributions

All authors were involved in drafting the article or revising it critically for important intellectual content and approved the final version to be published. CS had full access to all of the data in the study and takes responsibility for the integrity of the data and the accuracy of the data analysis. PE and CS conceived and designed the study. PE, PW, and CS acquired the data and analyzed and interpreted the data.

## Conflict of Interest Statement

The authors declare that the research was conducted in the absence of any commercial or financial relationships that could be construed as a potential conflict of interest.
